# Exo- and endoscopic two-step approach for anterior foramen magnum meningiomas: How I do it

**DOI:** 10.1007/s00701-025-06512-9

**Published:** 2025-04-14

**Authors:** Kenichiro Iwami, Yuichi Nagata, Tadashi Watanabe, Ryuta Saito

**Affiliations:** 1 Department of Neurosurgery, Nagoya University Graduate School of Medicine, 65 Tsurumai-Cho, Showa-Ku, Nagoya, 466 - 8550 Japan; 2 Department of Neurosurgery, Aichi Medical University, Aichi, Japan

**Keywords:** Meningioma, Anterior foramen magnum, Exoscope, Endoscope

## Abstract

Anterior foramen magnum (AFM) meningiomas are difficult to treat because they are situated in anatomically complex regions. One of the most common approaches for AFM meningiomas is the far lateral approach. We describe our exo- and endoscopic two-step approach (EETA) through suboccipital craniotomy. An exoscope is used to remove the tumour’s lateral part, whereas an endoscope is used to remove the residual tumour in the exoscope’s blind spots, including the brainstem’s ventral side. EETA is a viable option for treating AFM meningiomas because it provides an adequate surgical field of view and is minimally invasive.

**Supplementary Information:**

The online version contains supplementary material available at 10.1007/s00701-025-06512-9.

## Relevant surgical anatomy

The anatomy of the foramen magnum is complex, particularly in the anterolateral region where intricate bony components and neurovascular structures are present. Intradural foramen magnum meningiomas are classified based on their anatomical location with the dentate ligament as anterior, lateral, or posterior [1]. Anterior foramen magnum (AFM) meningiomas originate in the anterior aspect of the foramen magnum and frequently compress the brainstem and neurovascular bundle posteriorly. Consequently, posterior and posterolateral approaches provide limited visibility and manoeuvrability. The anterior transnasal approach provides direct access and is effective for lesions confined medially to bilateral hypoglossal canals. However, this approach risks cerebrospinal fluid (CSF) leakage and infection and is less suitable for lesions extending beyond the hypoglossal canal. The far-lateral approach, which involves partial condylectomy is used to remove AFM meningiomas from the posterolateral trajectory [2] but necessitates extensive skin incisions and drilling manoeuvres in deep and narrow surgical fields. Goel et al. reported that most AFM meningiomas could be resected using routine suboccipital craniotomy and microscopy [3]; however, small tumours required condylectomy because of the limited working space after tumour debulking. Gattozzi et al. classified AFM meningioma as type 2, 3, or 4 in the novel classification of foramen magnum meningiomas based on the relationship between the neurovascular bundle and tumour location [4]. They reported that type 2–4 tumours carry a high risk of permanent postoperative cranial neuropathy. Careful observation of the space anterior to the brainstem and neurovascular bundle, and meticulous tumour extension removal while preserving as much normal anatomical structure as possible can improve the rates of AFM meningioma resection and minimise surgical invasiveness.

### Description of the technique

**Fig. 1:**
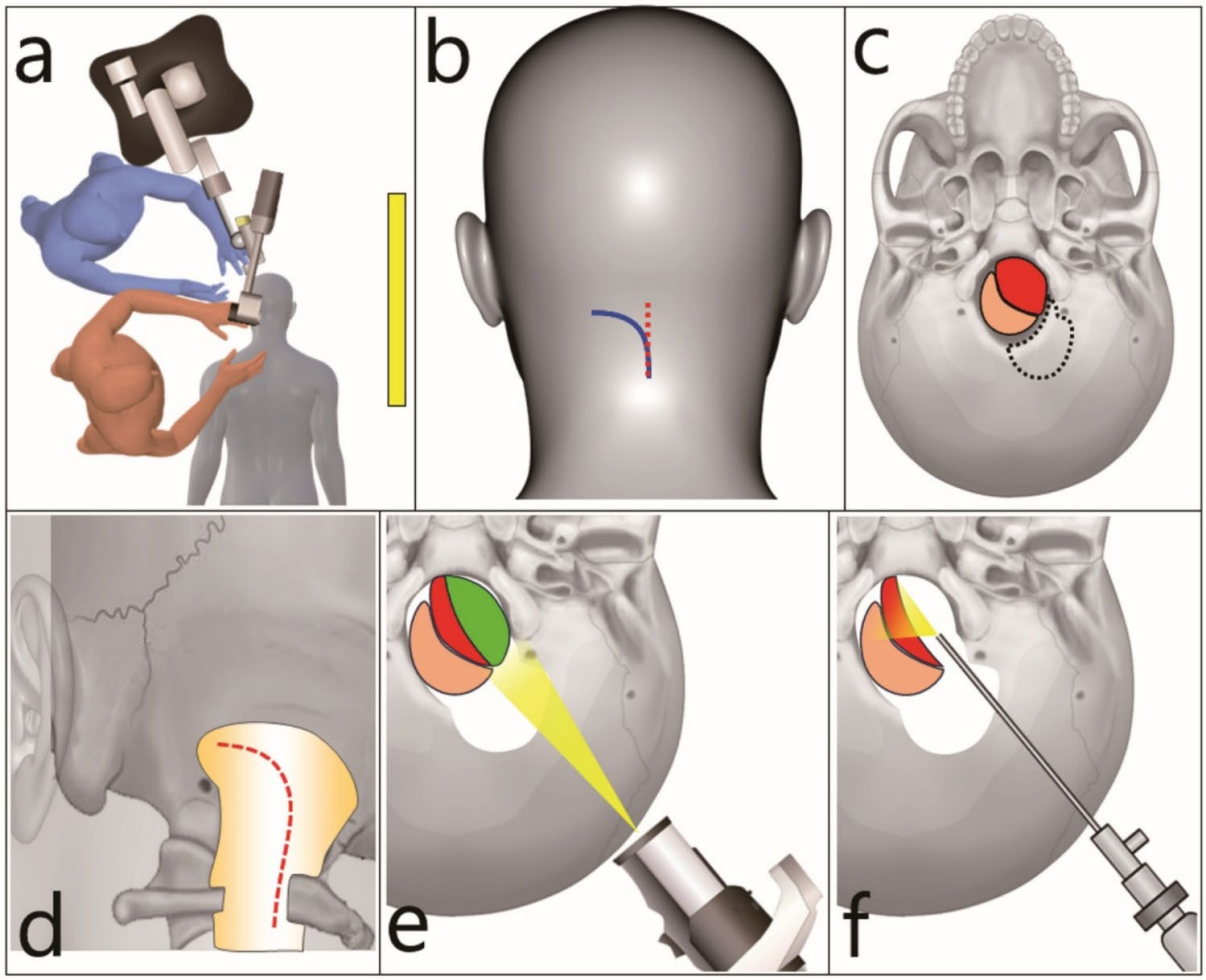
Schematic of exo- and endoscopic two-step approach (EETA). **a** Operating room layout. Red, operator; blue, assistant; yellow, surgical monitor. **b** Skin incision designs. Red dotted line, midline skin incision; blue line, C-shaped skin incision **c** Retrocondylar suboccipital craniotomy. Black dotted line, craniotomy; red, tumour; pale orange, brain stem **d** Dura incision design (red dotted line) **e** The first step of EETA. The lateral part (green) is removed using an exoscope. Red, tumour in the blind spot; pale orange, brain stem **f** The second step of EETA. The residual tumour (red) is removed using an endoscope. Pale orange, brain stem

Figure 1a shows our operating room layout. We always perform AFM meningioma excision surgery under continuous monitoring of motor-evoked potentials in the vocal cords and limbs. Patients undergo surgery in the prone position using a conventional suboccipital approach with a midline or C-shaped skin incisions (Fig. 1b). A retrocondylar suboccipital craniotomy is performed after atlas hemilaminectomy (Fig. 1c). We incise the dura in an arcuate fashion (Fig. 1d). The exo- and endoscopic two-step approach (EETA) for AFM meningiomas comprises two steps. First, the lateral part of the tumour is removed using an exoscope (ORBEYE; Olympus, Tokyo, Japan or VITOM3D; Karl Storz, Tuttlingen, Germany) through the space between the brainstem and condyle (Fig. 1e). Lateral part debulking provides additional working space for endoscope insertion. Second, the space anterior to the brainstem is assessed utilizing rigid endoscopes (0°, 30°, 45°, or 70°; outer diameter, 4 mm; Olympus, Tokyo, Japan or Karl Storz, Tuttlingen, Germany), and the residual tumour in the blind spots under the exoscope is removed (Fig. 1f). Curved surgical instruments are useful for approaching the contralateral deep surgical field in the anterior brainstem. Additionally, endoscopes can confirm haemostasis as they provide a clear view of the tumour resection cavity and the surrounding normal neurovascular structures while maintaining a clear field of view even under CSF presence. The dura, fascia, and subcutaneous tissue are sutured using absorbable sutures, and a bone flap is secured with a titanium plate whenever possible. Patients are typically required to remain in bed on the day of surgery, begin ambulation the following day, and are discharged within 5–7 days postoperatively. The EETA procedure for the two representative cases is as follows:

#### Case 1: Treated using a midline skin incision (Figs. 2 and 3 and Video 1)

**Fig. 2:**
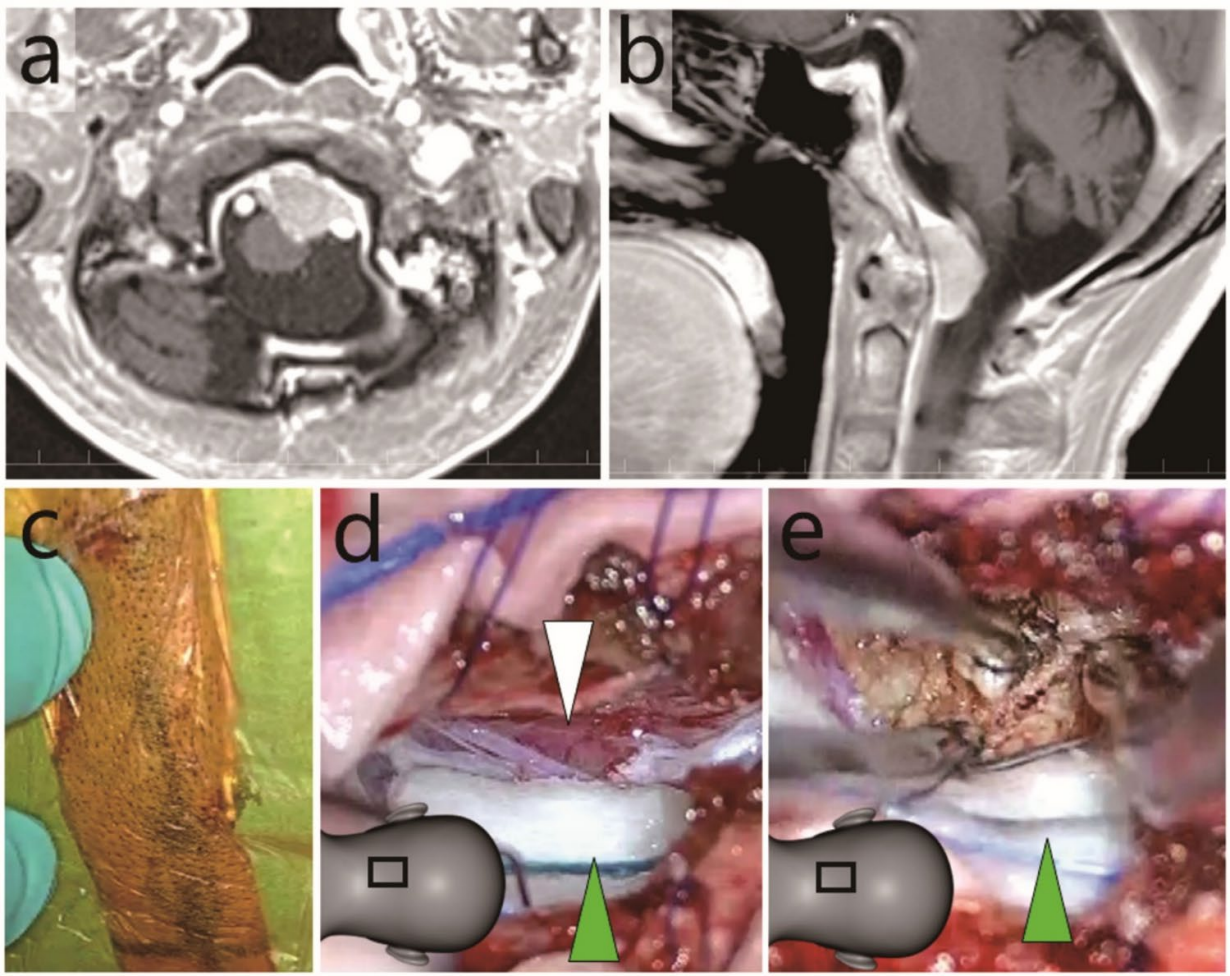
Case 1: Anterior foramen magnum meningioma resected through a midline skin incision **a** and **b** Pre-operative gadolinium-enhanced T1-weighted magnetic resonance imaging (**a**, axial; **b**, sagittal). **c** A 4-cm skin incision. **d** and **e** Intraoperative exoscopic photographs. The lateral part was exoscopically removed. Green arrowheads, brain stem covered with a cotton pattie; white arrowhead, tumour. The illustrations in panels **d** and **e** indicate the field of view

**Fig. 3:**
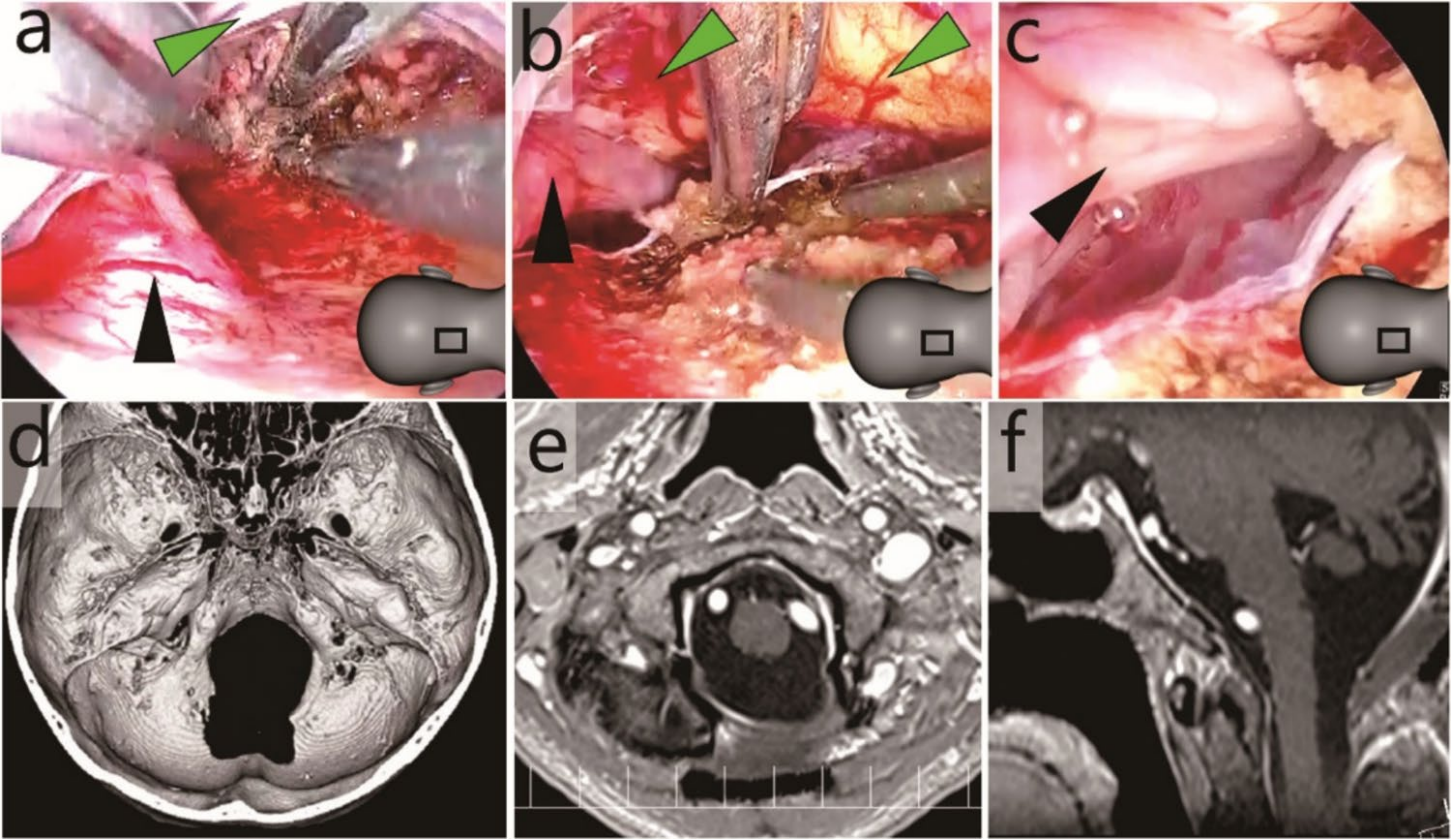
Case 1: Anterior foramen magnum meningioma resected through a midline skin incision. **a**-**c** Intraoperative endoscopic photographs. Green arrowheads, brain stem; black arrowheads, vertebral artery. The illustrations in panels a-c indicate the field of view. **a** and **b** The residual tumour in front of the brain stem was endoscopically removed. **c** Haemostasis was confirmed while the surgical field was flushed with artificial cerebrospinal fluid. **d** Post-operative three-dimensional computed tomography. **e** and **f** Post-operative gadolinium-enhanced T1-weighted magnetic resonance imaging (e, axial; f, sagittal)

Case 1 involved an AFM meningioma excised via a small midline skin incision (Fig. 1b, red dotted line). Owing to the restricted approach from the posterolateral direction, a smaller proportion of tumours could be exoscopically removed, and a higher proportion of tumours could be endoscopically removed.

#### Case 2: Treated using a C-shaped skin incision (Figs. 4 and 5 and Video 2)

**Fig. 4:**
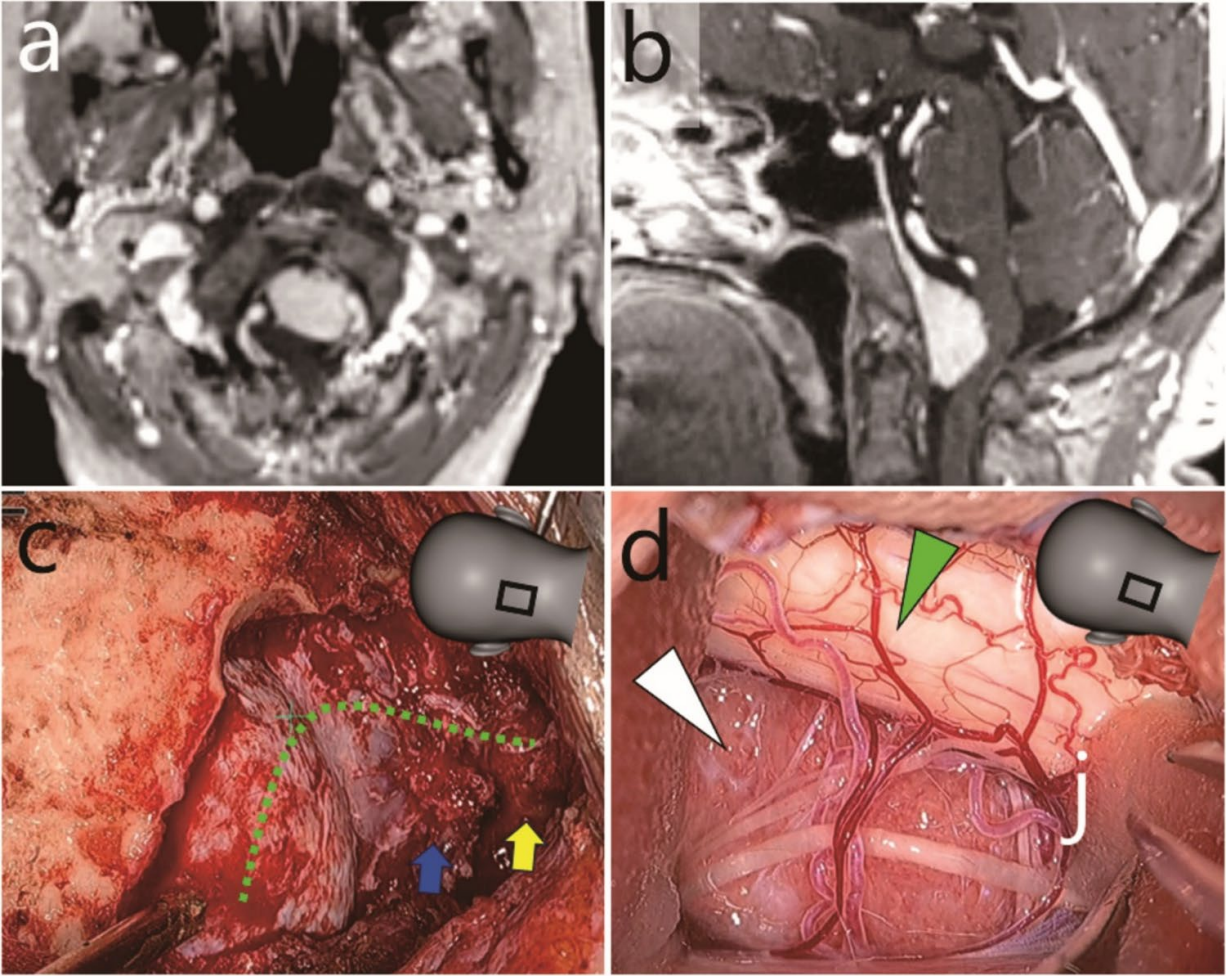
Case 2: Anterior foramen magnum meningioma resected through a left C-shaped skin incision. **a** and **b** Pre-operative gadolinium-enhanced T1-weighted magnetic resonance imaging (a, axial; b, sagittal). **c** and **d** Intraoperative exoscopic photographs. **c** Dura incision design (green dotted line). Blue arrow, foramen magnum; yellow arrow, atlas. **d** Lateral part of the tumour (white arrowhead). Green arrowhead, brain stem The illustrations in panels c and d indicate the field of view

**Fig. 5:**
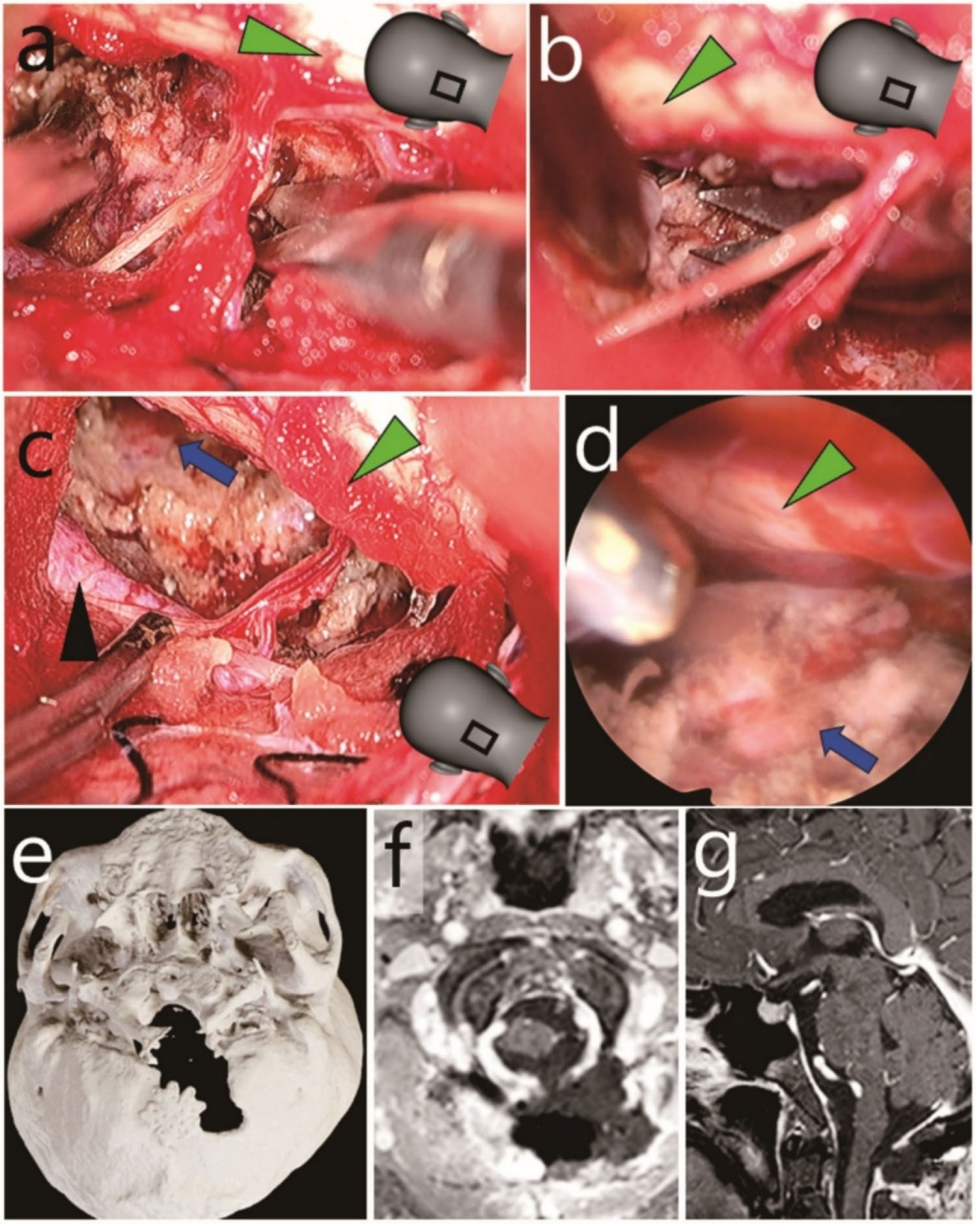
Case 2: Anterior foramen magnum meningioma resected through a left C-shaped skin incision. **a**-**c** Intraoperative exoscopic photographs. Majority of the tumour was exoscopically removed. Green arrowheads, brain stem; black arrowhead, vertebral artery; blue arrow, median of lower clivus. The illustrations in panels a-c indicate the field of view. **d** Intraoperative endoscopic photograph. Total resection and haemostasis were confirmed while the surgical field was flushed with artificial cerebrospinal fluid. Green arrowhead, brain stem; blue arrow, median of lower clivus. **e** Post-operative three-dimensional computed tomography. **f** and **g** Post-operative gadolinium-enhanced T1-weighted magnetic resonance imaging (f, axial; g, sagittal)

This was a case of an AFM meningioma excised via a left C-shaped skin incision (Fig. 1b, blue line). The majority of the tumour was removed in Step 1 given that the space anterior to the brainstem could be exoscopically observed from the posterolateral direction. However, endoscopy in Step 2 was still useful for coagulation of tumour attachment and haemostasis in the deep surgical field.

An endoscope provides a view to a deep and narrow surgical field, although an exoscope can be replaced with a microscope. The smaller the skin incision, the more limited the field of view from the lateral direction in Step 1. The reasons for using an exoscope are: 1) surgical monitor sharing with endoscopy, 2) ease of replacement with endoscopy, 3) three-dimensional (3D) image sharing, and 4) superior ergonomics [5–7].

### Indications

The EETA is used for AFM meningiomas because of its adequate surgical field observations and low invasiveness. The primary indication is a relatively small meningioma with minimal brainstem displacement. In such cases, the tumour’s deep portion remains challenging to exoscopically or microscopically observe even after debulking the lateral portion using the conventional suboccipital approach [4]. The EETA is well-suited for tumours not firmly adhered to the brainstem or vertebral artery because of the challenge in performing delicate dissection or complex suturing procedures under endoscopy.

### Limitations

Large (> 3 cm) and hard tumours are unsuitable given the EETA’s craniotomy size of approximately 2.5 cm. Additionally, easily bleeding tumours are unsuitable; therefore, we assessed the abundant vessels around the tumour on pre-operative contrast-enhanced computed tomography (CT) as having a high bleeding risk. Although pre-operative embolisation and piecemeal resection can manage this problem in selected cases, expert experience is required to efficiently perform such procedures. AFM meningiomas can be approached using the suboccipital approach under a microscope, without requiring an endoscope in cases with lateral displacement of the brainstem.

### How to avoid complications

Pre-operative examination of the blood vessels around the foramen magnum and throughout the posterior cranial fossa helps prevent inadvertent surgical bleeding, post-operative ischaemia, and venous congestion. Although contrast-enhanced CT is sufficient, angiography can provide better outcomes. Pre-operative imaging of the tumour location and neurovascular structures can help in predicting and preventing post-operative complications [3]. We aimed for delicate manipulation to preserve the brainstem, lower cranial nerves, and vertebral arteries. Additionally, careful haemostasis and dural closure prevent post-operative bleeding and CSF leakage.

### Specific information for the patient

General surgical risks such as infection and bleeding may be observed. Additionally, the lower cranial nerves can be damaged during tumour dissection, which can result in shoulder paralysis, dysphagia, dysphonia, and tongue paralysis.

### Ten key-point summary

1. EETA is a less-invasive surgical method for resecting AFM meningiomas.
2. The main indications for EETA are small-sized tumours with minimal brainstem displacement.
3. A small suboccipital craniotomy is performed following an atlas hemilaminectomy.
4. Removal of the lateral part of the tumour via an exoscope is the first step.
5. Debulking of the lateral part provides additional working space for endoscope insertion.
6. The second step involves examining the space anterior to the brainstem using an endoscope.
7. The residual tumour in the blind spots under the exoscope is removed using an endoscope.
8. Endoscopes are useful for confirming haemostasis in deep surgical fields.
9. Delicate manipulation to preserve neurovascular structures can prevent complications.
10. Although the exoscope used in the first step can be substituted with a microscope, the use of an endoscope is preferable for examining the space anterior to the brainstem.

## Supplementary Information

Below is the link to the electronic supplementary material.

Supplementary file1 (MP4 61838 KB)

Supplementary file2 (MP4 210037 KB)

## Data Availability

No datasets were generated or analysed during the current study.

## Abbreviations

AFM: Anterior foramen magnum
EETA: Exo- and endoscopic two-step approach
CSF: Cerebrospinal fluid
3D: Three-dimensional
CT: Computed tomography
MRI: Magnetic resonance imaging
